# The Role of Cardiac Magnetic Resonance in Myocardial Infarction and Non-obstructive Coronary Arteries

**DOI:** 10.3389/fcvm.2021.821067

**Published:** 2022-01-17

**Authors:** Kate Liang, Eleni Nakou, Marco Giuseppe Del Buono, Rocco Antonio Montone, Domenico D'Amario, Chiara Bucciarelli-Ducci

**Affiliations:** ^1^Department of Cardiology, Bristol Heart Institute, University Hospitals Bristol and Weston NHS Trust, Bristol, United Kingdom; ^2^Bristol Medical School, Translational Health Sciences, University of Bristol, Bristol, United Kingdom; ^3^Royal Brompton and Harefield Hospitals, Guys' and St Thomas Hospitals NHS Trust, London, United Kingdom; ^4^Department of Cardiovascular and Pulmonary Sciences, Catholic University of the Sacred Heart, Rome, Italy; ^5^Department of Cardiovascular Medicine, Fondazione Policlinico Universitario A. Gemelli IRCCS, Rome, Italy; ^6^Faculty of Life Sciences and Medicine, School of Biomedical Engineering and Imaging Sciences, King's College London, London, United Kingdom

**Keywords:** MINOCA, myocardial injury, myocardial ischemia, non-obstructive coronary arteries, cardiac MRI, myocarditis, Takotsubo syndrome

## Abstract

Myocardial Infarction with Non-Obstructive Coronary Arteries (MINOCA) accounts for 5–15% of all presentations of acute myocardial infarction. The absence of obstructive coronary disease may present a diagnostic dilemma and identifying the underlying etiology ensures appropriate management improving clinical outcomes. Cardiac magnetic resonance (CMR) imaging is a valuable, non-invasive diagnostic tool that can aide clinicians to build a differential diagnosis in patients with MINOCA, as well as identifying non-ischemic etiologies of myocardial injury (acute myocarditis, Takotsubo Syndrome, and other conditions). The role of CMR in suspected MINOCA is increasingly recognized as emphasized in both European and American clinical guidelines. In this paper we review the indications for CMR, the clinical value in the differential diagnosis of patients with suspected MINOCA, as well as its current limitations and future perspectives.

## Introduction

Myocardial infarction with non-obstructed coronary arteries (MINOCA) accounts for about 5–15% of all patients presenting with acute myocardial infarction (MI) referred for coronary angiography (1–5). The definition of MINOCA has evolved over time, initially used as an umbrella term encompassing various diagnosis such as MI with angiographic evidence of non-obstructive coronary arteries, acute myocarditis and TakoTsubo Syndrome, whilst currently the definition of MINOCA includes just myocardial injury due to an ischemic mechanism. Thus, whilst a working diagnosis of MINOCA at the time of angiography presumes the absence of coronary artery disease (CAD), the presence of unobstructed coronary arteries mandates further testing to determine a definitive diagnosis. Non-ischemic causes of ACS and unobstructed coronary arteries also necessitate further testing to identify a final diagnosis and CMR provides a unique diagnostic opportunity.

## Definition, Diagnosis and Clinical Relevance of MINOCA

MINOCA is defined by evidence of MI according to the “Fourth Universal Definition of MI” (6) with unobstructed coronary arteries on angiography (defined as stenosis severity of ≤50% in major epicardial coronary arteries), in the absence of any alternate diagnosis for the clinical presentation and/or myocardial injury (i.e., sepsis, pulmonary embolism, myocarditis, tachyarrhythmias, and hypertension) (7, 8). Importantly, MINOCA does not represent a single clinical entity but a heterogenous group of coronary artery disease etiologies, including coronary plaque rupture or erosion, coronary spasm (epicardial and microvascular), distal coronary embolism/thrombosis and spontaneous coronary artery dissection (SCAD) (9, 10).

The absence of obstructive coronary disease should not falsely reassure clinicians, and further testing is recommended aimed at identifying the underlying etiology of the clinical presentation to guide appropriate clinical management.

The role of invasive and non-invasive imaging as part of diagnosis of MINOCA has evolved with both strategies implemented. A recent review by Occhipinti et al. (9) proposes two alternative, and in part complementary, diagnostic pathways to confirm a diagnosis of MINOCA: one invasive using coronary angiography with OCT and one non-invasive using CMR. CMR is an important non-invasive imaging tool which provides comprehensive information on cardiac structure and function with the additional merits of non-invasive myocardial tissue characterization (i.e., presence and location of reversible and irreversible myocardial damage). The presence, extent and pattern of myocardial edema, inflammation, scar and fibrosis can be accurately identified by CMR allowing the confirmation of MINOCA, or the identification of underlying diagnosis with valuable prognostic information (11).

Patients with MINOCA have a better prognosis compared to patients with Myocardial Infarction with obstructive CAD (MI-CAD), but this is still associated with a lower survival rate of healthy individuals matched for age and sex (5, 12). Patients with MINOCA are generally younger at presentation than those presenting with MI-CAD, thus leading to an increased probability of future disability and premature exit from the workforce (13). Accurate identification of MINOCA, or alternative diagnosis in patients presenting with myocardial injury and unobstructed coronary arteries, has a substantial impact on both acute and chronic clinical management and patients' outcomes.

## Role of CMR in MINOCA

Invasive strategies for diagnosing MINOCA such as optical coherence tomography (OCT) have an important role in current diagnostic guidance. However, invasive strategies can pose a relative risk and its routine use may be limited by limited access and expertise. CMR offers a low-risk and radiation-free diagnostic strategy, it is readily available with only minor limitations and can also provide functional and structural data. CMR allows to differentiate between “true” MINOCA and MINOCA “mimics” with high sensitivity and specificity. A recent large observational study from Dastidar et al., demonstrated that CMR performed in patients with MINOCA at a median of 37 days after presentation identified a cause for the dynamic troponin rise in 74% of patients (11). The most frequent diagnoses were MI (25%), myocarditis (25%) and cardiomyopathy, including Takotsubo syndrome, (25%), with 25% of patients reported to have a “normal” CMR. These results confirmed on a large scale numerous previous smaller studies (14–16). When CMR was performed within 2 weeks the diagnostic yield increased to 84%, due to the ability to detect transient and reversible myocardial changes (myocardial hyperemia/inflammation/edema), which normally resolve after a few weeks, and therefore no longer detectable on a CMR performed several weeks, or months after the acute event (17).

The late gadolinium enhancement (LGE) technique (images acquired after contrast injection) is crucial in determining if myocardial damage is ischemic or non-ischemic. In ischemic etiologies, the contrast agent accumulates predominantly in the subendocardium or transmurally, reflecting the pathophysiology of the ischemic-necrotic wave-front phenomenon (partial thickness/subendocardial damage in shorter ischemic times, full thickness/transmural in longer ischemic times), as widely validated in histological studies both in animal and human studies (18, 19). Different are the patterns of LGE seen in non-ischemic differentials, such as myocarditis, which present with enhancement in the intra-myocardial or subepicardial layers. Parametric mapping (T1 and T2 mapping) also provides quantitative information in the involvement and burden of disease, particularly in non-ischemic etiologies (particularly relevant for excluding other cardiac pathologies when diagnosing MINOCA on CMR).

### CMR in International Guidelines to Date

Both the European Society of Cardiology (ESC) and the American Heart Association (AHA) have recognized the central role of CMR in the diagnostic work up of patients with MINOCA. In particular, the updated 2020 ESC guidelines for management of patients with non-ST elevation MI (7) indicates that a CMR is recommended (Class 1B) in all MINOCA cases with no obvious cause.

Previously, the AHA determined that CMR is the only non-invasive imaging test that is recommended to investigate these patients further following invasive angiography (8).

## MINOCA: Atherosclerotic and Non-Atherosclerotic Causes

**Figure 1:**
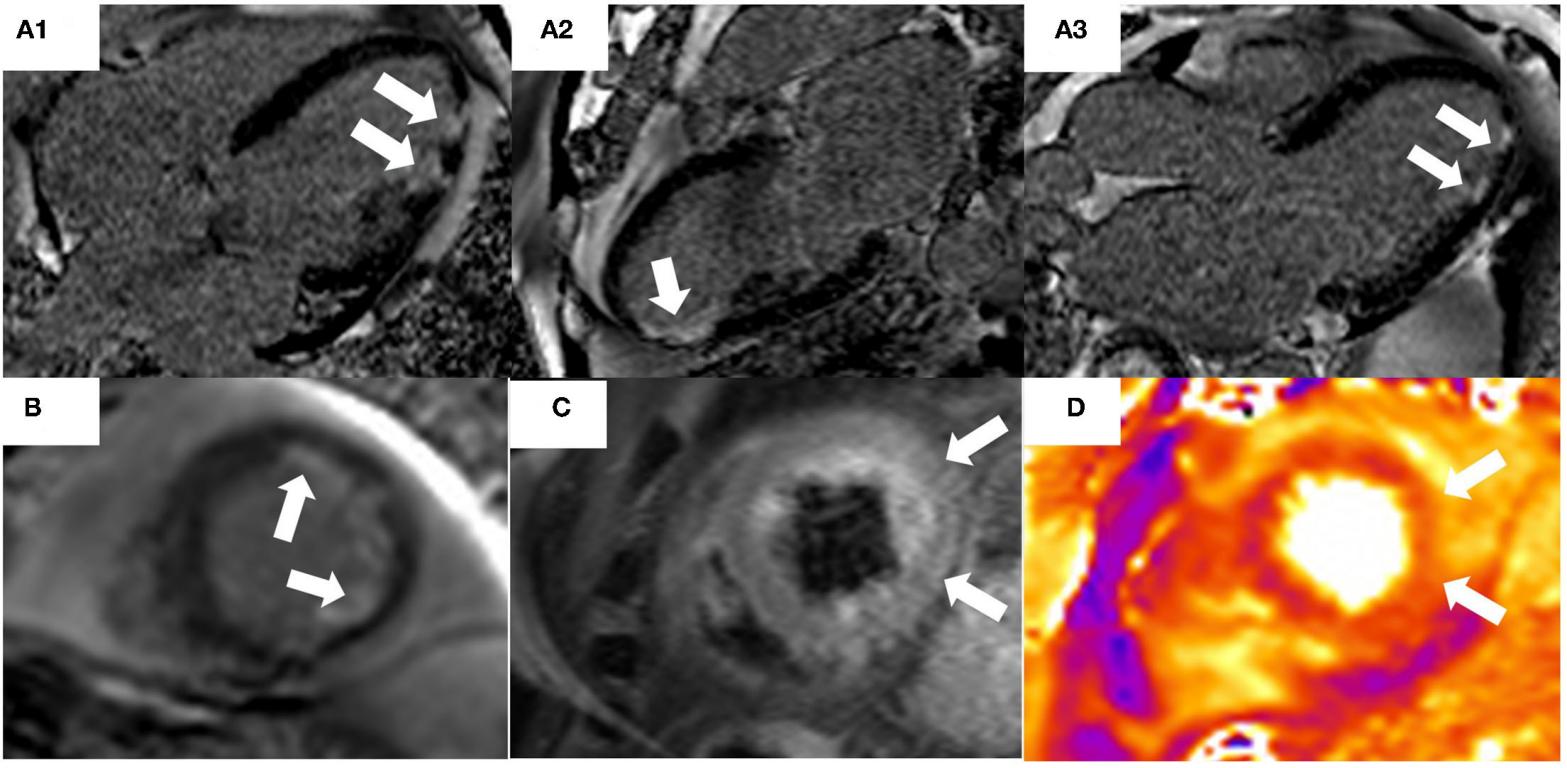
74-year old male presented with chest pain and troponin rise with lateral T-wave inversion on electrocardiogram. Invasive coronary angiography demonstrated non-obstructed coronary arteries. CT pulmonary angiography was also negative for pulmonary embolus. A Cardiac MRI demonstrated evidence of myocardial infarction with typical ischemic pattern of subendocardial late gadolinium enhancement (LGE) [white arrows, **(A1–A3)**: 4 Chamber, 2 Chamber, 3 Chamber, **(B)** short axis] in the apical inferior and lateral segments. Areas of high signal on T2-STIR imaging **(C)** and T2-mapping **(D)** correlating to the area of infarction from LGE imaging and the ECG abnormalities, suggesting acute myocardial edema due to acute myocardial injury.

CMR findings are similar in patients with MINOCA in comparison to those with MI-CAD (Figure 1). Multi-modality imaging and an integrated approach should be considered in all cases where the final diagnosis remains unclear.

### Atherosclerotic Causes

Atherosclerotic causes of MINOCA include unstable non-obstructive plaques (plaque rupture or plaque erosion) with spontaneous autolysis of the associated intracoronary thrombus. Typical angiographic appearances in these cases are of haziness, or small filling defects within the coronary lumen (20). Intracoronary imaging such as intravascular ultrasound (IVUS) and OCT are important in these cases. However, availability and expertise for IVUS and OCT may not be readily offered in all centers, especially in the acute setting. In such cases, CMR may be beneficial in seeking a final diagnosis.

### Non-atherosclerotic Causes of MINOCA

Non-atherosclerotic causes of MINOCA include epicardial and microvascular coronary spasm, SCAD and distal coronary embolism/thrombosis.

Prolonged episodes of epicardial vasospasm can result in MINOCA, with the frequency reported as high as 46% of patients presenting with an NSTEMI and no culprit lesion (21). However, spontaneous episodes may not be documented at the time of coronary angiography and an invasive provocative testing is often required to establish the diagnosis (8). SCAD may appear as non-obstructive coronary artery disease on angiography because of gradual tapering of the vessel that may only be recognized after careful review of the angiogram, or by intracoronary imaging (22). In patients with distal coronary embolism/thrombosis the source of thromboembolism may be a hypercoagulable state (e.g., Factor V Leiden, protein C and S deficiency, and anti-phospholipid syndrome), partial lysis of an epicardial coronary thrombus with distal embolization, or a distal embolism from another non-coronary source (e.g., paradoxical embolism with an intra-cardiac shunts) (8). Echocardiography (transthoracic and transesophageal) may be used for the search of possible sources of coronary embolization (e.g., infective endocarditis, intra-cardiac thrombi and/or shunts.) Microvascular spasm is another cause of MINOCA, but its real prevalence is unknown. In patients with suspected coronary vasomotor disorders, intracoronary Acetylcholine (ACh) provocative testing is the diagnostic tool of choice as it may elicit an increased microvascular hyperconstrictive response (23–25).

CMR findings in both atherosclerotic and non-atherosclerotic presentations of MINOCA are similar as those in obstructive ACS with occlusive plaque rupture: an LGE pattern either subendocardial or transmural would be indicative of a myocardial infarction. If T2 weighted imaging is also performed, these images would be able to indicate myocardial oedema due to the acute damage.

In a study by Reynolds et al. enrolling fifty women, plaque disruption was found as cause for MINOCA in 38% (16/42) of cases using IVUS (20). CMR performed in these patients demonstrated subendocardial or transmural LGE associated with elevated myocardial signal intensity on T2-weight imaging consistent with myocardial edema. This pattern correlated with the vascular territory identified on IVUS and was distinct from non-ischemic causes of myocardial injury (26, 27).

It should be noted that the absence of LGE/scar does not necessary exclude the diagnosis of MINOCA but this may represent an aborted infarct, likely due to spontaneous recanalization and cessation of permanent myocardial damage leading to scar seen on LGE imaging. However, areas of myocardial edema are often present which support the occurrence of temporary cessation of flow/myocardial stunning (28).

## MINOCA Mimics

MINOCA differential diagnoses include a variety of diseases which are important to recognize.

### Acute Myocarditis

Myocarditis is an inflammatory disease of the myocardium. It can manifest in various ways but can often present with troponin positive chest pain; it is the most commonly presenting non-ischemic differential in the diagnostic work up for MINOCA. In its acute form, symptoms may range from mild dyspnea or chest pain to fulminant heart failure, life-threatening arrhythmias and cardiac death (29). The most common precipitants are viruses, but with other non-viral causes. A previous episode of myocarditis may be diagnosed incidentally on CMR when investigating for other cardiac presentations such as etiology of severe left ventricular (LV) dysfunction. The gold-standard investigation for definitive tissue diagnosis of myocarditis remains an endomyocardial biopsy (EMB) which, while in experienced hands is relatively low risk, is an invasive procedure and available in limited centers (30).

Use of CMR to diagnose myocarditis is a safe and reliable alternative to EMB, utilizing the unique capability of CMR for non-invasive tissue characterization and is more readily available. Within the most recent ESC (31) and AHA guidelines (32), use of CMR is recommended in investigating and diagnosing myocarditis. Furthermore, the number of those diagnosed with myocarditis is higher when CMR is utilized as part of investigation for the work up of MINOCA (33, 34), which has subsequent implications on ongoing clinical management. The Updated Lake Louise Criteria (2018) (35) provides a comprehensive set of diagnostic criteria to assist the diagnosis of myocarditis using all the various CMR imaging markers with the indicated cut-off values for the interpretation of normal/abnormal, including myocardial signal intensity relative to the skeletal muscle and elevated T1 and T2 mapping values. Parametric mapping was an additional criterion in the updated version, incorporating this technique as part of clinical guidelines rather than solely within the research domain. Supportive criteria such as pericardial effusion and LV wall motion abnormalities are also included within the diagnostic criteria.

**Figure 2:**
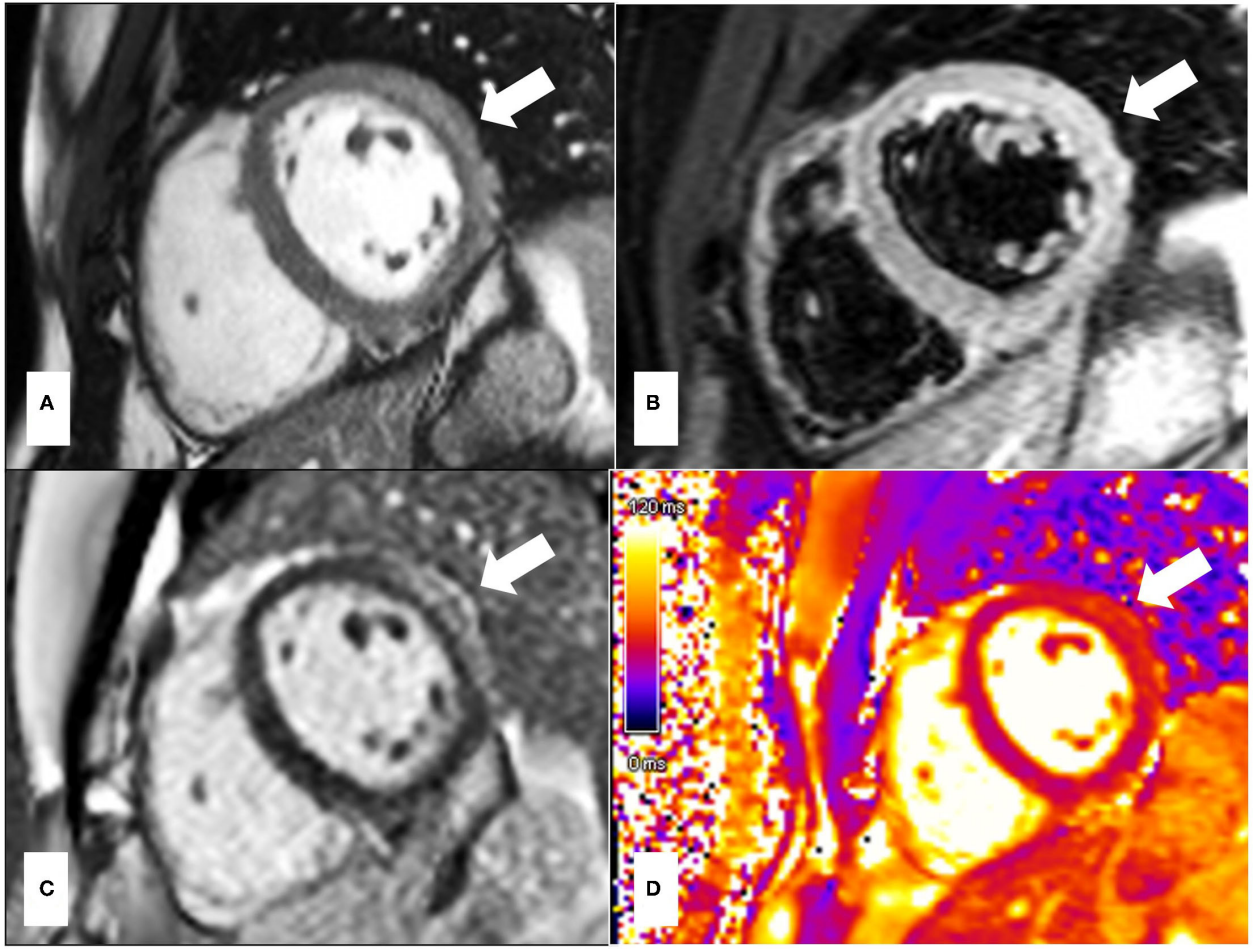
33-year-old presented with troponin positive chest pain and inferior ST elevation on ECG. He reported recent flu-like symptoms following mRNA COVID-19 vaccination. **(A)** is the post-contrast steady state free precession (SSFP) cine showing a bright signal intensity in keeping with myocardial oedema due to acute myocardial injury, which is also in keeping with the T2-STIR imaging **(B)** and T2 mapping **(D)** images which demonstrate high signal intensity in the mid lateral wall correlating with subepicardial late enhancement on gadolinium imaging **(C)**. The pattern of myocardial injury noted is in keeping with acute myocarditis. The areas of abnormalities are indicated by white arrows.

The CMR patterns observed in myocarditis typically include intra-myocardial or subepicardial LGE, distinct from the subendocardial or transmural enhancement seen in ischemic etiologies. In the acute setting, extensive myocardial oedema can be accompanied by a transitory increased in wall thickness (Figure 2).

### Takotsubo's Syndrome

Takotsubo's Syndrome (TTS) is an acute form of systolic heart failure, often triggered by a physical or emotional event, in the absence of a culprit epicardial coronary obstruction (36). LV regional wall motion abnormalities typically extend beyond the territory subtended by a single epicardial coronary artery with a circumferential (non-epicardial coronary) pattern. The classically described anatomical variant is “apical ballooning,” which is characterized by apical hypo/akinesia and basal compensatory hyperkinesia. It should be noted that other variants may have a reversed pattern affecting the basal segments, or the mid LV segments, although these are far less common. Clinical presentation and electrocardiographic (ECG) findings are similar to those of patients presenting with MI-CAD making coronary angiography necessary to exclude obstructive CAD.

Different diagnostic criteria for Takotsubo's syndrome have been proposed although a definitive consensus remains unresolved. The most commonly used are the Revised Mayo Clinic Criteria (37) and the recently introduced diagnostic criteria proposed by the European Society of Cardiology (InterTAK Diagnostic Criteria) (38). Historically, TTS and myocarditis were included among the causes of MINOCA, however, since they are considered non-ischemic causes of myocardial injury, they should be considered separately.

CMR is recommended in all patients with suspected TTS as it allows exclusion of other causes of myocardial injury. In addition, assessment of biventricular function and visualization of regional contractility can be made to aide diagnosis, often seen (but not always performed) on ventriculogram during invasive coronary angiography. Identification of possible cardiac complications associated with TTS can also be made on CMR, and more clearly than on echocardiogram, particularly myocardial wall rupture and ventricular thrombi (39).

Patients with Takotsubo's syndrome often display transmural myocardial edema on T2-weighted imaging associated with the dysfunctional segments (38). The amount of myocardial edema seen in acute TTS often causes an acute increase in LV mass, which can also be observed as increased wall thickness (40). Since myocardial edema is a transient and reversible phenomenon, usually resolving between 3 and 6 months post the index event, its identification is dependent on timing from presentation to CMR; earlier imaging will likely yield higher diagnostic accuracy (41). More novel parametric mapping techniques provide a more objective and quantitative assessment of myocardial edema (42). However, cut-off values of native mapping have not been established in this setting.

**Figure 3:**
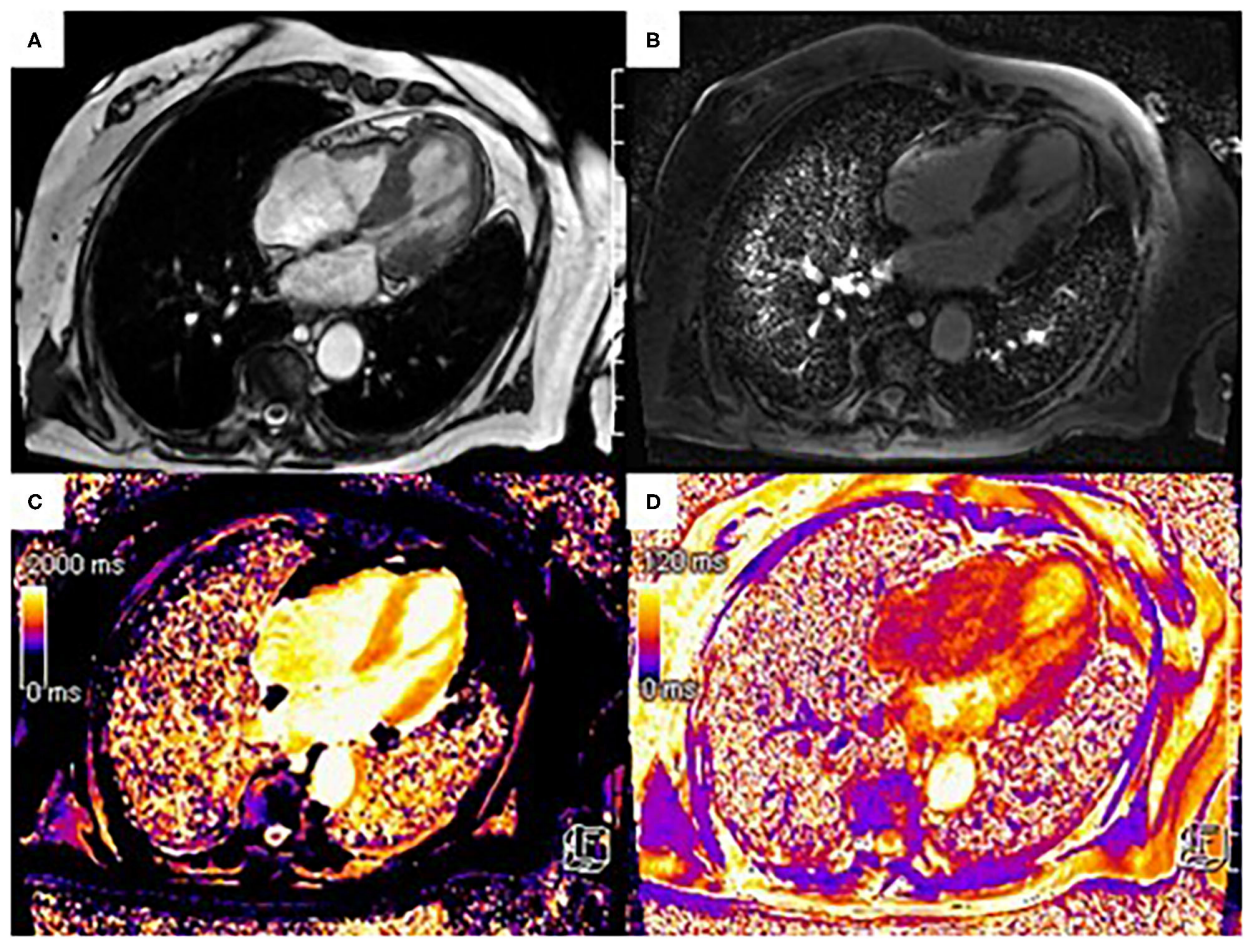
90-year-old patient presenting to the emergency department with chest pain, ST segment elevation in the anterior leads at electrocardiogram and elevated troponin levels. Urgent coronary angiography excluded obstructive coronary artery disease. Three Tesla cine Steady-State Free Precession sequence (SSFP) CMR showed akinesis of the apical segments with hypercontractility of the basal segments **(A)**, absence of LGE **(B)** and elevated native T1 mapping [1,400–1,550 msec (local reference range: 1,086–1,292 msec)] **(C)** and elevated T2 mapping values [50–55 msec (local reference range: 30–45 msec)] **(D)** in correspondence with the area of apical akinesia. Based on the clinical context and imaging findings, the patient was diagnosed with apical ballooning in the context of Takotsubo syndrome.

The use of early gadolinium enhancement sequences (EGE) is important in identifying intra-cavity thrombus as a complication of impaired left ventricular function, seen as low signal intensity areas adjacent to the dysfunctional segments (39). The absence of LGE is often pathognomonic in those presenting with TTS, distinct from MI-CAD and myocarditis. Areas of high signal intensity on LGE imaging may be mistaken for ischemic scar, but often represents areas of acute myocardial oedema. Follow up CMR scans in these cases will demonstrate resolution of myocardial edema but also confirm the absence of LGE (Figure 3). Rarely, LGE may have a transmural location at the hinge points between the akinetic and the hypercontractile segments, but this is as result of shearing forces rather than ischemia (36, 39).

### Other Findings

Other myocardial disorders, including inherited cardiomyopathies, may mimic presentation of MINOCA and it is important to understand their distinguishing features on CMR.

#### Hypertrophic Cardiomyopathy

Common CMR findings in patients with hypertrophic cardiomyopathy include increased signal in T2-weighted imaging suggestive of myocardial edema secondary to ischemic changes in the context of the microvascular dysfunction, and inducible myocardial hypoperfusion, with or without the presence of intramyocardial LGE. The presence of myocardial edema and myocardial perfusion defect in association with the presence of LGE is a significant prognostic indicator of ventricular arrhythmias (43, 44). In addition, CMR can more accurately measure LV wall thickness in comparison to echocardiography, with additional presence of intra-myocardial LGE in the hypertrophied segments. T1 mapping values may also be elevated.

**Figure 4:**
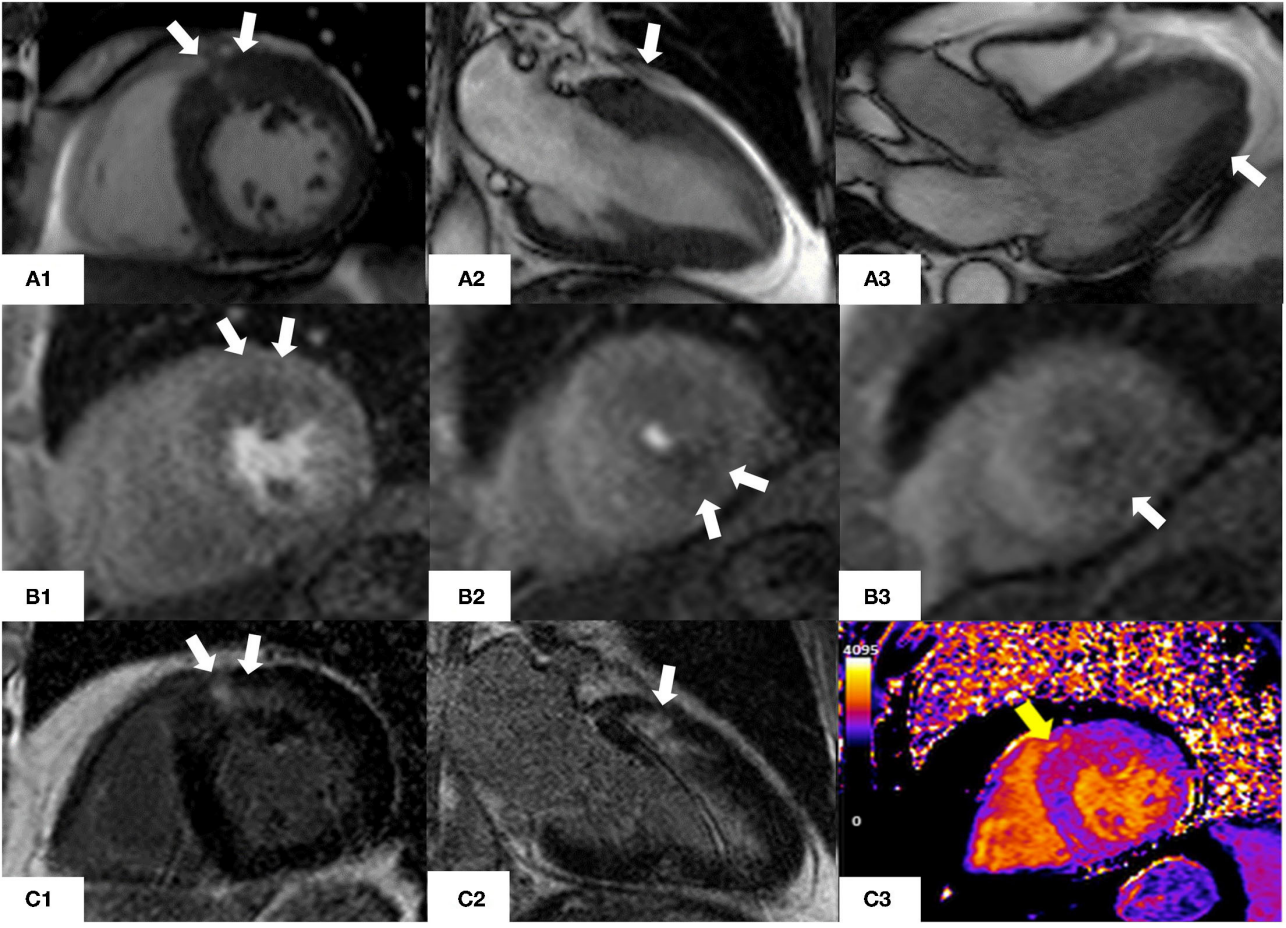
CMR findings in a 76 year old man with asymmetric hypertrophic cardiomyopathy who presented with atypical chest pain and non-sustained ventricular tachycardia on a recent ECG-Holter. **(A1–A3)** Steady-state free precession sequence (SSFP) cine imaging showing asymmetrical hypertrophy of the basal anterior wall and mild apical hypertrophy (white arrows). **(B1–B3)** Stress perfusion images showing inducible microvascular dysfunction of the hypertrophic basal anterior wall and mid-cavity and apical inferior wall (white arrows). **(C1, C2)** Late gadolinium enhancement imaging (LGE) showing patchy myocardial fibrosis of the hypertrophied segments (white arrows). **(C3)** Native T1 mapping (basal short axis view). Increased native T1 values (yellow arrow) in the basal anterior and basal anteroseptal walls (1,170 msec, normal range 1,000 ± 50 msec) matching the patchy LGE noted in panels **(C1, C2)**.

Figure 4 shows CMR findings in a patient presenting with angina and mild increase in the levels of troponin T.

#### Dilated Cardiomyopathy

Approximately 25% of patients with dilated cardiomyopathy have evidence of intramyocardial fibrosis (45), which is an independent prognostic factor beyond LV ejection fraction (LVEF) (46). There is evidence that diffuse fibrosis assessed by T1 mapping is significantly predictive of all-cause mortality and heart failure events (47). In addition, stress and rest myocardial blood flow is decreased in segments with LGE, indicating microvascular dysfunction in the LV segments with fibrosis (48). This could potentially be the pathophysiological component of those with a presentation of suspected MINOCA in those with underlying dilated cardiomyopathy. The clinical relevance of these findings has not yet been proven.

#### Pericarditis

Acute pericarditis often mimics acute coronary syndrome due to the similar presentation, the presence of ischemic ECG abnormalities and the possible concomitant troponin elevation. In those that have a more marked troponin rise and angiographically normal coronary arteries, CMR may be helpful to differentiate between myopericarditis, characterized by the extension of pericardial inflammation to the myocardium despite a preserved LV systolic function; and perimyocarditis, characterized by a decrease in LV systolic function (49). Patients with pericarditis or myopericarditis are treated with anti-inflammatories rather than anti-platelet therapy. Common CMR findings in patients with pericarditis include thickening of the pericardial layers and pericardial effusion detected on T1-weighted CMR, or cine imaging while edema of the inflamed pericardium is visualized on T2-weighted imaging associated with pericardial enhancement on LGE sequences (50). Involvement of the myocardium in pericarditis cases will present with intra-myocardial +/− subepicardial LGE.

#### Cardiac Amyloidosis

In cardiac amyloidosis, a characteristic abnormality of myocardial, blood-pool gadolinium kinetics and global subendocardial LGE are common (51). It is characterized by an early subendocardial LGE, and later transmural LGE, associated with abnormal gadolinium kinetics that may manifest as simultaneous myocardial and blood nulling or suboptimal myocardial nulling, pathognomonic of cardiac amyloidosis. Importantly, parametric mapping plays an important diagnostic and prognostic role in cardiac amyloidosis with T1-mapping demonstrating higher sensitivity in detecting early disease compared to conventional T1-weighted sequences (52); Extracellular volume (ECV) also plays an important role in guiding and monitoring therapies and prognosis (53–55).

#### Cardiac Sarcoidosis

Cardiac sarcoidosis is a systemic granulomatous disease which may present with chest pain, ischemic ECG changes and ventricular arrhythmias. Common CMR features include septal thinning, ventricular dilatation, systolic dysfunction and pericardial effusions. A non-ischemic pattern of LGE is seen, commonly affecting anteroseptal and inferolateral walls, although other segments can also be involved, including the right ventricle (56, 57). T2-weighted imaging plays an important role in monitoring the activity of cardiac sarcoidosis by detecting edema associated with inflammation vs. quiescent disease (58). Importantly, the presence of LGE is recognized as a risk factor of mortality and ventricular arrhythmias even in patients with preserved ejection fraction (59), while the absence of LGE is a negative predictor associated with low risk of adverse cardiac events, even in cases with severely impaired LVEF (60).

**Figure 5:**
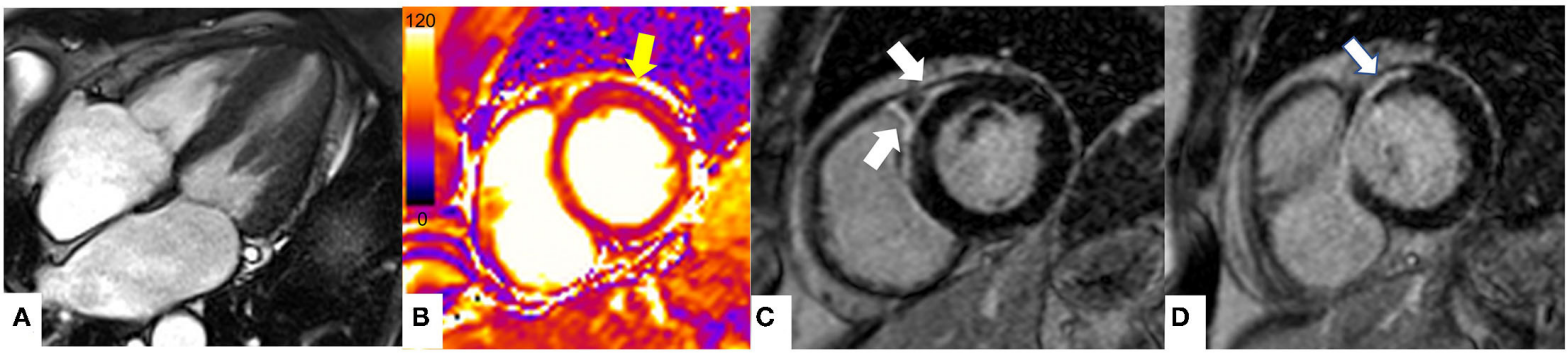
The role of CMR in non-ischemic cardiomyopathies mimicking MINOCA. CMR findings in a patient with known cardiac sarcoidosis presenting with chest pain. **(A)** Steady state free precession sequence (SSFP) cine sequence 4 chamber (end-systole) showing preserved ventricular systolic function **(B)** T2 mapping (basal segments) showing high T2 values (arrow) in the basal anterior wall (T2 = 62 ms, normal values T2 < 55 ms at 1.5 T) suggestive of myocardial oedema. **(C, D)** Late gadolinium enhancement imaging (LGE) at the level of basal segments demonstrating prominent subepicardial to mid wall enhancement in the basal anteroseptum and anterior wall extending into the adjacent right ventricular anterior wall (arrows). The pattern of myocardial injury noted is in keeping with acute cardiac involvement in sarcoidosis.

Figure 5 shows the CMR findings of systemic diseases with cardiac involvement presenting with possible MINOCA.

#### Normal CMR Findings

The etiology of MINOCA could be identified in 85% with the combination of intracoronary OCT imaging and CMR (20). Although CMR is a robust tool in the working up of MINOCA, there are reports that a wide range of MINOCA presentations (8–67% of MINOCA cases) have no evidence of wall motion abnormalities, myocardial edema or LGE15, (61, 62), causing a diagnostic dilemma for clinicians. A study showed that 25% of patients presenting with MINOCA had plaque disruption detected by intravascular ultrasound (IVUS) despite normal CMR findings (20). Microembolization or coronary spasm inducing minimal increase in troponin levels (63), or a shorter duration of ischemic injury not detectable at CMR. In fact, while CMR can detect even down to 1 gram of infarcted myocardium, low levels of myocyte necrosis can still pass undetected (28, 64). At the same time, a normal CMR appearance may be the result of a broader spatial distribution of necrosis in patients with type two MI, which may not generate the same level of myocardial insult.

Nonetheless, in these such cases CMR plays an important role in identifying areas of infarction that could be missed if further downstream testing is not performed after angiography demonstrates unobstructed coronaries.

## Limitations of CMR

Whilst CMR is has increasingly become a part of routine cardiac diagnostics for some time, there remains limitations to its use. CMR is still perceived as an expensive and time-consuming test. A recent cost-analysis using a hypothetical population of patients with acute myocardial injury and angiographically normal coronaries demonstrated that CMR guided treatment led to cost reductions in the medium to long-term, offsetting the initial cost and so its perceived high cost may not be as limiting as widely thought (65). Although the technology available, both in terms of physical hardware and software has progressed, there is the advent of more novel techniques such as parametric mapping (35) and rapid cine sequences (66), which should be extended to more centers. An increasing use of focused and rapid sequences may reduce scanning times, but experienced radiographers and clinicians are still required to provide guidance and support patient flow.

Expertise for CMR often remains in larger referral centers but the educational opportunity to train in CMR have expanded in the last few years.

Although scanning patients with legacy (non-conditional MRI) devices has been shown to be safe under the right circumstances (67), susceptibility artifacts remain a problem for all pacing devices and may render some images diagnostic. Appropriate referral and choice of those for whom a CMR is likely to yield diagnostic benefit is as important as being able to scan those with devices previously deemed contraindicated.

Gadolinium-chelated based contrast agents (GBCAs) used in CMR scanning are renally excreted and have previously been avoided in those with stage IV-V renal impairment. A recent systematic review and meta-analysis has demonstrated good safety data of Group II GBCAs in these patients with a very low risk of 0.07% developing nephrogenic systemic fibrosis (68). The older linear, non-cyclical GBCAs are almost obsolete in their use given their comparative safety data to newer agents.

The diagnostic yield of CMR is highest in the first 2 weeks from the acute presentation (11), and timely access to CMR would be ideal.

## Future Directions

### Parametric Mapping in Clinical Practice

CMR relaxometry techniques have the unique property of visualization and quantification of myocardial abnormalities, independent of whether myocardial disease is focal or diffuse; and during subclinical stages of disease, compared to conventional CMR imaging. Importantly, T1- and T2-mapping quantification has been included in the revised guidelines for the diagnosis of myocarditis using CMR (35). It should be noted that in the Stockholm Myocardial Infarction with Normal Coronary Arteries study 2 (SMINC-2), 77% of all patients with MINOCA received a diagnosis when investigated early compared to SMINC-1 study where only 47% received a definite diagnosis by CMR (69). However, it is not clear if the improved diagnostic accuracy noted in SMINC-2 study was due to a more comprehensive protocol or earlier imaging. If such techniques and early access are available, then this would be favorable.

### Strain-Encoded Magnetic Resonance

Strain-Encoded Magnetic Resonance (SENC) is an advanced tagging technique that further quantifies myocardial “strain” or deformation analysis (70). Myocardial deformation is particularly useful in the clinical setting of assessment of viability and regional myocardial function but with additional benefits in non-ischemic pathologies such as cardiomyopathies, diabetic heart disease and cardiotoxicity and characterizing myocardial disease early (71). This is particularly relevant and is supplementary to standard CMR techniques in correctly identifying MINOCA and its mimics to ensure accurate diagnosis and downstream management. Furthermore, contrast administration is not required for myocardial strain, or SENC. Whilst SENC in acute myocardial infarction with culprit lesions has been investigated (72, 73), the use of SENC specifically in MINOCA patients has not yet been validated and requires further investigation.

### Cardiac Magnetic Resonance Fingerprinting and Quantitative Techniques

Cardiac Magnetic Resonance Fingerprinting (MRF) simultaneously maps multiple CMR properties such as T1, T2 and proton density in the myocardium (74) and was initially introduced in mapping the brain tissue (75, 76). An important advantage of MRF over the conventional T1- and T2-mapping is that it does not rely on complete recovery of magnetization and consequently provides more accurate and reproducible T1- and T2-quantification (77). Additionally, it provides information regarding tissue characterization beyond T1 and T2 values, such as perfusion, diffusion, T2^*^ and ECV without the administration of exogenous contrast agents (77). Cardiac MRF has been used in a small group of cardiac transplant recipients and there was good agreement between cardiac MRF T1 values and T1 measurements acquired by conventional T1 mapping (78). However, further investigation is required for the validation of this new technique in the working diagnosis of MINOCA.

### Rapid Scanning Protocols in Clinical Practice

Rapid CMR protocols have been developed for 1.5 T scanners, particularly in the assessment of cardiomyopathies and chronic ischemic heart disease. Scan times can be reduced, maximizing efficiency and scanning capacity without compromising diagnostic accuracy (79). Whilst tested in developing countries to facilitate access to CMR, its use more globally should be considered, particularly if it enables easier access as part of diagnosis of MINOCA. Scans can be performed within 30 min, with an average time as short as 18 min for contrast studies for the evaluation of ventricular function and fibrosis. Importantly, the advent of rapid CMR protocols can alter clinical management in up to 56% of patients investigated with CMR (66). It is an area of growing interest but its use in the work-up of MINOCA needs to be validated in large clinical trials.

## Conclusion

CMR plays a crucial role in the diagnosis of MINOCA. It can provide comprehensive and accurate diagnosis of a broad range of cardiac pathologies and differentiate between the multiple pathologies that mimic a diagnosis of MINOCA (80). Exclusion of other non-ischemic etiologies responsible for the acute clinical presentations is prognostically important and enables accurate, patient centered care. Whilst limitations remain, there has been increasing progress in novel techniques, accessibility of CMR and adoption of CMR within clinical practice and guidelines, and its role is proving to be increasingly valuable in this cohort of patients.

## Author Contributions

All authors listed have made a substantial, direct, and intellectual contribution to the work and approved it for publication.

## Funding

RM was supported by a research grant from the Italian Ministry of Health (Ricerca Finalizzata GR-2019-12370197).

## Conflict of Interest

CB-D was the CEO (part-time) of the Society of Cardiovascular Magnetic Resonance and has received speakers fees from Circle Cardiovascular Imaging, Bayer and Siemens Healthineers. The remaining authors declare that the research was conducted in the absence of any commercial or financial relationships that could be construed as a potential conflict of interest.

## Publisher's Note

All claims expressed in this article are solely those of the authors and do not necessarily represent those of their affiliated organizations, or those of the publisher, the editors and the reviewers. Any product that may be evaluated in this article, or claim that may be made by its manufacturer, is not guaranteed or endorsed by the publisher.
